# Disaster Response in Italian Nursing Homes: A Qualitative Study during the COVID-19 Pandemic

**DOI:** 10.3390/geriatrics7020032

**Published:** 2022-03-17

**Authors:** Barbara Plagg, Giuliano Piccoliori, Adolf Engl, Christian J. Wiedermann, Angelika Mahlknecht, Verena Barbieri, Dietmar Ausserhofer, Peter Koler, Sara Tauber, Manuela Lechner, Walter A. Lorenz, Andreas Conca, Klaus Eisendle

**Affiliations:** 1 Institute of General Practice and Public Health, Provincial College for Health Professions Claudiana, 39100 Bolzano, Italy; giuliano.piccoliori@am-mg.claudiana.bz.it (G.P.); adolf.engl@am-mg.claudiana.bz.it (A.E.); christian.wiedermann@am-mg.claudiana.bz.it (C.J.W.); angelika.mahlknecht@am-mg.claudiana.bz.it (A.M.); verena.barbieri@am-mg.claudiana.bz.it (V.B.); dietmar.ausserhofer@claudiana.bz.it (D.A.); klaus.eisendle@sabes.it (K.E.); 2 Faculty of Education, Free University of Bolzano Bozen, 39100 Bolzano, Italy; 3 Department of Public Health, Medical Decision Making and HTA, University of Health Sciences, Medical Informatics and Technology, 6060 Hall in Tyrol, Austria; 4 Nonprofit Organization Forum Prevention, 39100 Bolzano, Italy; koler@forum-p.it (P.K.); sara-tauber@hotmail.com (S.T.); manuelalechner71@hotmail.com (M.L.); 5 Department of Applied Social Sciences, Faculty of Humanities, Charles University, 182 00 Prague, Czech Republic; walter.lorenz@unibz.it; 6 Department of Psychiatry, Bolzano Central Hospital, 39100 Bolzano, Italy; andreas.conca@sabes.it; 7 Academic Teaching Department of Dermatology, Venereology and Allergology, Bolzano Central Hospital, 39100 Bolzano, Italy

**Keywords:** nursing home care, disaster management, disaster preparedness, infection prevention and control measures, prevention

## Abstract

Nursing homes (NHs) have been among the care settings most affected by both the virus itself and collateral damage through infection protection and control measures (IPC). However, there is a paucity of research regarding disaster response and preparedness of these institutions. The present study aimed to analyze disaster response and management and to develop prospective strategies for disaster management in NHs. A qualitative survey including (i) residents, (ii) nursing staff, (iii) relatives of residents, and (iv) NHs’ medical leads was performed. Data were collected by 45 in-depth interviews. Our results indicate that the shift from resident-centered care towards collective-protective approaches led through the suspending of established care principles to an emergency vacuum: implementable strategies were lacking and the subsequent development of temporary, immediate, and mostly suboptimal solutions by unprepared staff led to manifold organizational, medical, and ethical conflicts against the background of unclear legislation, changing protocols, and fear of legal consequences. IPC measures had long-lasting effects on the health and wellbeing of residents, relatives, and professionals. Without disaster preparedness protocols and support in decision-making during disasters, professionals in NHs are hardly able to cope with emergency situations.

## 1. Introduction

The worldwide spread of SARS-CoV-2 triggered an urgent, ongoing public health crisis beginning in early 2020. Older adults have been disproportionately impacted by COVID-19, with higher susceptibility to severe illness, hospitalization, and death [1,2]. Worldwide, nursing homes (NHs) have been at the epicenter of the COVID-19 pandemic with high mortality rates due to the residents’ susceptibility to infection and the congregate nature of NHs with multiple vectors for infection [3]. However, elderly people do not only belong to the SARS-CoV-2 risk group, but also to those who suffer increased morbidity and mortality as a result of the withdrawal of social interaction and mental stimulation due to infection protection and control (IPC) measures [4]. Accordingly, NHs have been among the care settings most affected by both the virus itself and collateral damage through IPC measures. Since NHs were exposed to long-standing issues such as staff shortages, poor resources, and regulatory gaps [5,6] before the pandemic, the COVID-19 crisis became a major stress test for these care institutions.

COVID-19 led to a sudden and unprepared interruption of supporting relationships and dynamics, as well as daily routines, and forced all groups involved—professionals, residents, and relatives—to change existing behaviors and strategies while flexibly adapting to the emergency situation. However, while institutional care represents an important public health issue as they provide accommodation and long-term care for millions of people worldwide, there is a paucity of research regarding disaster response and preparedness of these institutions [6,7]: to date, only limited literature exists with regard to NHs during a public health crisis and more research is needed, including the often neglected perspectives of residents, NH staff, and informal caregivers [8]. This seems especially important since the implementation of shielding measures in NHs are of ongoing discussion and frail NH populations represent a vulnerable target for future disasters.

Overall, it is of high public health importance to elucidate disaster responses, challenges, and strategies of NH staff, since care workers (CW) are valuable disaster responders and their experiences and knowledge needs to be included in the development of disaster protocols [9,10]. Additionally, the perspective and needs of residents and their relatives as informal caregivers and important resources in daily care should be incorporated into disaster management plans [11].

Therefore, we performed a comprehensive qualitative survey in Northern Italy between September 2020 and March 2021 including four different subgroups: (i) residents, (ii) nursing staff, (iii) relatives of residents/informal caregivers, and (iv) GPs in the role of the NH’s medical lead. The aim of the study was to analyze:

(i) Disaster response and management including immediate strategies and challenges in NHs;
(ii) The effects and impacts of the IPC measures on residents, relatives, and professionals;
(iii) Prospective strategies for disaster management in NHs.

## 2. Materials and Methods

### 2.1. Design

Due to the novelty of the situation, we conducted explorative qualitative research. Based on our purpose, the grounded theory was chosen as the methodological angle to address our research question as the subject of our study is emergent, requiring the greatest possible openness to explore changes in, as well as antecedents, linkages, and consequences of the organization, the maintenance of participants’ health, and strategies of disaster management in a systematic manner an inductive approach, since previous knowledge of the phenomenon of interest is insufficient or fragmented [11,12]. The present study was the first phase of a multi-phase study aiming to better understand disaster response during the pandemic in NHs. This paper is based on the qualitative data collected through 45 individual interviews between September 2020 and March 2021. The study period thus refers to the time of the second wave up to the development and use of the vaccine as an IPC measure. Triangulation and analysis of data were carried out within the first half of 2021.

### 2.2. Study Population

For a comprehensive analysis of different perspectives, we included 4 groups in our study and conducted a total of 45 interviews in 24 different NHs to develop patterns, concepts, categories, and dimensions of the phenomena. Overall, our cohort included (i) care workers of different professional levels, (ii) GPs (medical leads of NHs), (iii) residents, and (iv) relatives of NH residents. Participants were recruited from 79 NHs in the northernmost province of Italy, Bolzano, with a catchment area of 531,178 predominantly German-speaking inhabitants.

Following the sequential and flexible, yet systematic, guidelines of grounded theory, the ongoing analysis of the data material allowed for targeted recruitment. Thus, the selection of participants was based on an iterative process that sought to maximize the richness of the research data until thematic saturation was reached [13]. In particular, we sought the inclusion of participants from different NHs, German and Italian speaking people, in urban and rural areas, and from NHs affected by COVID-19 and without COVID-19 cases until December 2020. Out of 45 interviews, we conducted 14 interviews with NH staff, 6 interviews with residents, 16 interviews with relatives, and 10 interviews with GPs. Four out of the fourteen care workers were in a leading position as directors of nursing (DON). Following informed consent, semi-structured interviews were conducted.

### 2.3. Data Collection

With regard to the fragmentary data situation regarding disaster response in NHs, the research team prepared an interview guide with open-ended questions for each group (available on request). The questions focused on the time span from the outbreak of the pandemic to the time of the interview. A pretest was conducted to evaluate the interview guide with one person from each of the four target groups. Subsequently, the researchers adjusted the interview outline according to pilot interviews. Following consent, data collection was performed with individual semi-structured face-to-face interviews with all 45 participants by experienced qualitative and bilingual researchers. Only residents able to understand the purpose of the study and to provide informed consent were included. The interviews were held in private rooms with only interviewers and interviewees present, and the time ranged from 15 to 90 min. All interviews were carried out by researchers with expertise in qualitative research. All interviews were audio recorded and transcribed with the transcription software f4 (Version: 7.0.6, Marburg, Germany), generating 637 pages of raw data.

### 2.4. Data Analysis

After preparation of the raw data files and data cleaning, and rigorous and systematic reading, the open and axial coding of the de-identified transcripts allowed major themes to emerge from raw data. Specific text segments were labeled to create categories, which were subsequently conveyed into upper- and lower-level key themes and processed into a framework. To ensure reliability, the interviews were coded independently by three researchers, which showed high consensus. Continuous revision and refinement of the category system was carried out within the team to identify subtopics, contradictory points of view, new insights, and to ensure that analytical deductions were congruent with the extracts. Due to the large dataset, we used the analysis software MAXQDA (Release 20.2.2, Berlin, Germany) for analysis and triangulation of data by comparing the responses and codes between the four subgroups.

### 2.5. Ethics Approval

Ethical approval was obtained from the board of the institution where lead researchers were based (Fachhochschule für Gesundheitsberufe Claudiana, Institute of General Practice and Public Health, Bolzano, Italy) on 16 June 2020. Additionally, approval was obtained from the management team of all included NHs. All participants signed a written informed consent to participate in the study. They could withdraw their participation from the study at any point in time. All residents were cognitively able to understand the written information about the study and to give informed consent. Data security was ensured as all individuals with access to the raw material were bound to data secrecy. All data on study participants were anonymized to protect their identity. The datasets generated and analyzed during the current study are available from the corresponding author on reasonable request.

## 3. Results

### 3.1. Sample Description

A total of 37 women (80%) and 9 men (20%) participated in the survey. Four interviews were conducted in Italian, with the rest in German. The interviewees were between 21 and 89 years old (mean: 55 years; Table 1 and Table 2).

### 3.2. Main Themes and Sub-Themes

In response to the increasing spread of SARS-CoV-2, residential homes and NHs in Italy and other countries were gradually isolated with very little contact to the external environment. Parallel to the closure, the daily activities changed due to IPC measures. As a consequence, residents of NHs lost the autonomy to choose their everyday activities, interactions, and movements by themselves. While the measures were handled and implemented differently and thus varied between NHs, three meta-themes overarching all NHs and target groups became evident: as a consequence of the lockdown measures, it came to a sudden (i) suspension of established care principles, (ii) ethical dilemmas, and (iii) isolation (Table 3).

### 3.3. Main Theme 1: Suspending of Established Care Principles

#### 3.3.1. Sub-Theme: Lack of Strategies

Parallel to the disruption of most established care activities and principles under the emergency regulation, immediate solutions for addressing core needs and key principles of care were lacking and a structural vacuum of disaster management strategies became evident. Consequently, strategies had to be improvised by the staff themselves against the background of unclear legislation, changing protocols, and fear of legal consequences.

*“A specific strategy would have been needed immediately for NHs. That was certainly missing.”*

*(GP 04)*

*“We didn’t have one [a protocol].”*

*(CW 14)*

*“There is no guideline, so what do we do now? […] Now everyone is doing a bit [of] what they think is right. Of course, that’s not helpful to calm people down, to develop a strategy.”*

*(GP 24)*

Due to the strong ethical foundation in the health care profession and the perceived commitment to care values by health professionals, the implementation of the measures created manifold conflicts of conscience among nursing staff and GPs, who were moving in the field of tension between collective IPC measures and individual patient-centered care. The disruption of established care values in favor of rigid IPC strategies left an “emergency vacuum”, where finding acceptable compromises became a major challenge for professionals, especially for GPs and DONs in charge, who had to take responsibility for their decisions.

*“It’s a risk–benefit trade-off. A very difficult balancing act. It’s stressful […] because the possibilities were limited and you didn’t really know what to do, how to decide. Living with uncertainty was very difficult, very stressful and is not yet completely over.”*

*(GP 20)*

Within our interviews, it became evident that mandatory emergency guidelines were adapted by professionals based on their own ethical considerations and according to the given possibilities (i.e., spatial–infrastructural dimensions) of a structure, leading to a heterogeneous situation in the different NHs—often perceived as unfair and arbitrary by relatives.

Parallel to the collapse of “old” care principles and routines and the absence of relatives and volunteers as care support, a number of new work tasks had to be implemented by NH staff, including measuring temperature, disinfecting people and rooms, assisting with meals in the rooms, establishing contacts via telephone/internet with relatives, procuring materials, organizing and accompanying visits, and controlling distance and hygiene rules. Many of the new tasks had little in common with the intrinsic care mandate and were experienced as burdensome.

*“The staff […] refused to bring the residents into this room. It was one room, all separated by plexiglass. It was heartbreaking […] the relatives also burst into tears and many said, [it’s] like being in prison.”*

*(CW 37)*

#### 3.3.2. Sub-Theme: Immediate Strategies

While no participant underestimated the danger of the virus or fundamentally questioned IPC measures, nursing staff actively looked for compromise strategies in between infection control and proven care principles. Accordingly, finding a “new normal” included creative ways to bridge the care deficiencies arising from IPC measures, whilst trying at the same time to maintain the greatest possible safety from infections. Immediate strategies included finding ways for physical contact, distance visits, video calls, allowing relatives to have access to dying people, allowing residents to meet within the building or garden. To make this possible, legal frameworks had partly to be bypassed. However, measures—in particular visitor bans and isolation—were never infringed without establishing in-house rules such as wearing PPE for visiting relatives at the deathbed and time restrictions. The care of the dying turned out to be a particular ethical challenge: our results indicate that most of those in charge have not followed the guidelines of isolation in this regard anyway, since unaccompanied dying was generally considered a moral failing. On the other hand, those who have adhered to the measure and did not allow relatives to the deathbed, feel that they have supported a morally not justifiable measure.

*“We didn’t stick to the guidelines there either. We always embraced our residents.”*

*(CW 37)*

*“Nothing went well at all. […] No one could say goodbye.”*

*(CW 25)*

*“We didn’t stick to the guidelines, patients who were dying, who were seriously ill, were allowed to be visited with PPE.”*

*(GP 10)*

The complete ban on visits was, overall, difficult to maintain for months. Sooner or later, all NHs were forced to enable “distance visits”, i.e., interactions between the residents and their relatives and friends through windows, balconies, or gardens. Generally, relatives and residents actively searched for ways to stay in touch and professionals tried to find ways to enable interaction.

*“The visitor ban is something that absolutely does not work in NHs.”*

*(GP 36)*

*“Yes, they [the sons] were outside waving. […] I had to take it as it was.”*

*(Resident 18)*

However, some of the developed methods, such as visits behind an acrylic glass panel with phones on both sides or video calls, were found to be ambivalent. Depending on the underlying pathology (i.e., dementia, numbness, etc.), measures proved to be temporarily useful for some, while not working at all for others.

*“At the very beginning, with the Plexi […] that was a disaster. Better nothing than that, because […] that just irritated them, blinded them and made them really restless.”*

*(Relative 01)*

*“They came to visit me, one inside and one outside with the phone. […] But it wasn’t a phone call, we didn’t even hear each other. You can’t hear anything through the windows.”*

*(Resident 18)*

*“The video calls didn’t work because she couldn’t recognize me on the tablet.”*

*(Relative 01)*

Overall, due to the manifold pathologies, possibilities, and needs of nursing home residents, it was evident to professionals that measures had to be individually adapted. This, however, led to ethical concerns on how arbitrary restrictions can be loosened or not.

#### 3.3.3. Sub-Theme: Organization and Communication

While Italian NHs have reached a good quality of care, similar to the European average, the pre-pandemic system was already operating at the limit of economic survival, with no provision for an emergency [7]. As a consequence, during the pandemic the unprepared NH system met with serious organizational difficulties. Besides assisting residents with their daily activities and needs, the crisis posed a sudden additional organizational workload onto NH staff: internal and external communication massively increased and had to be reorganized, administrative workload increased, staff schedules had to be constantly adjusted, material resources such as PPE had to be procured, the interaction with relatives had to be changed to digital devices, residents with COVID-19 had to be isolated and treated, and COVID-19 testing had to be organized.

Long-lasting gaps in the organizational structure of NHs became quickly apparent. At the time of the outbreak of the pandemic, many NHs in the northernmost Italian region were without a medical director—a position usually filled in by a GP in Italy. This vacancy became a major problem, since during the emergency the medical lead position was no longer just a theoretical function, but an important position associated with responsibilities and decision-making tasks. Additionally, the staff shortage, an international issue of concern, came to head in the crisis. The high infection rate among nursing staff and the long quarantine periods at the beginning resulted in low patient-to-nurse ratios, which in turn had a negative impact on the care of the residents.

*“They saw that it’s [medical head of a NHs] not just a theoretical position, but one that has to be carried out. And with a lot of responsibility.”*

*(GP 23)*

*“Human resources is always the problem […]. In this situation, I need a lot more, I don’t have any, and of course I run the risk of people being overworked or falling ill. We were already at the limit before and now we are even more at the limit.”*

*(GP 24)*

Overall, professionals reacted with extreme flexibility to this period of emergency. In NHs with a good working atmosphere, positive dynamics among the health care staff were found: the exchange within the team was intensified, nursing staff showed at the beginning high motivation and willingness to stand in for absent colleagues, GPs frequently worked overtime, and digitization could be improved. However, in NHs with poor pre-pandemic working atmospheres, the crisis became an unmanageable stress test. Additionally, motivation and resilience among the professionals changed with the progression of the crisis. The initial enthusiasm to tackle the crisis was slowly replaced by feelings of exhaustion, critical attitudes with regard to IPC, chronic stress, and reduced willingness to take over for absent colleagues, volunteers, and informal caregivers.

*“I also noticed that it was good for the team, the cohesion. Even if the team was exhausted or burnt out.”*

*(CW 09)*

*“The willingness to step in was good at the beginning but then, over time… […] Bottom line: It’s always a question of time. If it takes forever, it becomes more and more problematic.”*

*(GP 24)*

During crisis events, communication is critical at all phases of disaster management. For NHs, external communication with other institutions proved to be difficult: general guidelines, emergency strategies, and contact persons were missing at the beginning of the pandemic. Within the course, recommendations and guidelines came from various services, institutions, and bodies located at the provincial and state level, but a distinct hierarchy, clear information flow, and competent points of reference were absent, leading to heterogeneous information flows within the different NHs. The unclear communication led to uncertainties among the professionals regarding the implementation of measures, while the differences in handling the measures between the NHs led to incomprehension on the side of relatives and residents. Communication between NHs themselves also proved to be suboptimal during the crisis, as well as communication with hospitals. Nursing and medical professionals within the NHs often felt that policies were not well thought through, and that they were left out of the communication loop. A welcome advance in the context of the emergency was the expansion of tele-health and telemedicine services to NHs.

*“We did not know where our patients were going, in which hospital, how they were doing. The flow of information was very deficient.”*

*(GP 20)*

Besides increased inter-professional exchanges, the pandemic has changed NHs’ communication practices with relatives. Overall, communication was entirely shifted from face-to-face communication to digital channels. It was challenging for NHs to introduce technology and tele-health approaches. Mainly, because NHs were lacking appropriate devices or did not have the knowledge to use them, because a designated person serving as point-of-contact for relatives and residents was missing, and because for many residents, digital communication was not possible due to their underlying condition. Despite the difficulties, however, approaches that had long been called for were implemented under the pressure of the pandemic. For example, medical prescriptions could be transmitted digitally. A positive perception of communication between the NHs and relatives was associated with (i) availability of telephone or e-mail enquiries, (ii) regular telephone calls between residents and relatives, (iii) timely passing on of information regarding the residents’ health status, (iv) transparent information about IPC measures, (v) empathetic communication, and (vi) reporting details of the residents’ everyday life. Here, a heterogeneous pattern emerged: while some NHs managed to maintain a positive exchange with relatives, some did not. Despite some relatives’ advocacy for change, there was little response to their actions, often leading to a feeling of disenfranchisement and lack of voice. The lack or insufficiency of communication proved to be a high burden throughout the whole pandemic and led to a decline in people’s trust in NHs and care institutions. On the other hand, positive communication led to a more coherent feeling and confidence that the NH staff were effectively managing the pandemic.

*“If we hadn’t called, hardly any information [came] from them.”*

*(Relative 31)*

*“That you could at least call. […] That was very helpful, that you could at least talk on the phone.”*

*(Relative 19)*

Where home care manager, GP, and DON agreed on principles of care and IPC, they made up a good ethical climate and succeeded in finding a shared attitude and handling of the situation. Where this was the case, the internal communication between the GP, the DON, and the employees of the NHs is described as dynamic and good as they adapted flexibly to the new situation and increased the exchange within the team. Besides inter-professional exchanges, however, communication between staff and residents as well as residents themselves massively decreased due to the isolation measures.

*“With isolated people, communication was zero, you could say. We tried to get the daily hygiene behind us as quickly as possible.”*

*(CW 37)*

Within the sub-category “material resources”, numerous codes on the availability of personal protective equipment (PPE), as well as disinfectants emerged. Despite the importance of these basic measures, in several NHs PPE equipment was lacking or had to be re-used over a long period of time. The lack of fundamental components to ensure the safety of the staff and the residents led to incomprehension and uncertainties at the professional side. However, there were also structures that had fewer difficulties because of their proximity to providers or because they had already had to deal with infections or multi-resistant germs in the past and had PPE in stock.

*“Because the employees often didn’t understand that, why aren’t we sufficiently protected?”*

*(CW 34)*

#### 3.3.4. Sub-Theme: Professional and Private Burden

Working under disaster conditions can have a major impact on responders. In the immediate aftermath of the outbreak, health professionals in NHs were at the front line of infection, had to overcome chaos and unclear regulations, whilst flexibly adapting to the medical and non-medical needs of the residents and practice self-preservation. Besides the burdensome balancing act between new principles and old care values, working conditions were exacerbated by unclear information flow, lack of knowledge, changing strategies, wearing of PPE, longer working hours, compensation for colleagues, and various anxieties.

It is known that care workers, as the largest groups of emergency responders during a disaster, are at risk of developing psychosocial problems that may need interventions [14]. During the pandemic, a wide variety of stressors came up on the professional side including the fear of infecting family members, who in turn could infect others and the fear of bringing the virus into the NHs. Additionally, pressure substantially increased on NHs, as GPs in charge obtained the information that sending residents to hospital was discouraged or outright refused.

*“I am a danger to my daughter or to my husband.”*

*(CW 33)*

Within their private sphere, NH workers felt confronted with social stigma by their surrounding community, due to their work with (possibly) infected patients and the negative media coverage regarding the situation in the NHs. Since health professionals are mostly female, another frequent concern was related to childcare and the question who will look after the offspring when schools or kindergartens are closed.

*“If my child stays at home, and I would have to work, but I can’t because I have no one else at home. So all of this really plays a role and impacts me at work too.”*

*(CW 08)*

Walking the tightrope between IPC measures and care duties led to hardly solvable ethical dilemmas and moral distress, which in turn led to psychosocial stress for many health care workers who felt unprepared for the psychosocial and mental challenges of a disaster. Overall, the reported symptoms included sleeplessness, sadness, depressive symptoms, and emotional breakdowns. While professionals generally expressed the importance and need for supervision and psychosocial support, it was only rarely offered in a few NHs. Interestingly, however, where psychosocial support was possible in the form of telephone calls, it was barely used. The reasons for this may be found in the digital form, time constraints, stigma, and too little awareness of the importance of the support offer.

*“I went home and I cried the whole morning because (…) not only did I not promote health, but I supported illness by not being able to give people something to drink when they were thirsty, by not being able to turn them when it was necessary to change them.”*

*(CW 33)*

However, as observed in previous studies [8], we found that NH staff activated various formal and informal resources as coping strategies to deal with emergency situations, including intuitive problem solving, a sense of staff unity, leading a healthy lifestyle, reduction of news consumption, social interactions and formal support through coaching, and private psychological counseling.

*“I saw a psychologist for a while and that did me good for a while. My resource is clearly my private life, where I have learned, thank God, to switch off.”*

*(CW 33)*

### 3.4. Main Theme 3: Ethical Dilemmas

#### 3.4.1. Sub-Theme: Self-Determination versus Community Welfare

Ethical dilemmas arise during decision-making processes, where a person has to choose between one or more options, neither of which is fully acceptable from an ethical perspective. While respect for patient autonomy has been the cornerstone of clinical bioethics for several decades and is a key principle in NH care, the individual is also an interdependent member of a community, where the right of self-determination must be limited by the welfare of others in that community [15]. Moreover, in non-pandemic times, many ethical challenges such as end-of-life-care, privacy, autonomy, informed consent, use of restraints, and offensive behavior exist on a daily basis in NHs [16]. Usually, they are solved according to the international consensus on key ethical principles of care in NHs including (i) accessible continuum of services on the basis of need, (ii) explicit focus on quality of care, (iii) quality of life for the residents of these facilities [17], and (iv) respect for the patient’s/resident’s autonomy [18]. However, the pandemic led to a disruption of ethical frameworks, since autonomy was challenged on multiple fronts, individual choice was limited, health services declined, and communal activities were suspended. Professionals felt left alone in balancing the resident’s right to autonomy and self-determination versus community health. Overall, as the isolation became more than a temporary measure, professionals perceived it as unethical not to include residents in the decision-making process and proactively searched for ways to respect their autonomy and dignity as much as possible.

*“How do you deal with that? […] On the one hand you want to help people and lock them up as little as possible, but what do we do if a corona case comes in? […] No one was actually able to help me. I had to look for a solution for myself.”*

*(GP 04)*

*“You should also* ask *the elderly what their needs are and to what extent they want to be protected. […] this is the target group for whom we are doing this, no one has asked them.”*

*(GP 23)*

*“At least in our NHs, the residents were never asked what they wanted.”*

*(CW 37)*

#### 3.4.2. Sub-Theme: Responsibility and Fear of Legal Consequences

Within the atmosphere of uncertainty, missing guidelines, and ethical dilemmas, professionals in NHs were left with the responsibility to make medical choices. An emerging sub-theme with regard to responsibility was the concern of having to face legal consequences because care activities might not have been legally correct.

*“Because for me, the mandatory measures are a guideline on the one hand, but on the other hand […] it is incomprehensible to me that a daughter cannot see her mother. […] But if it really goes wrong, then you are responsible, also legally.”*

*(GP 24)*

*“Then you ask and you get a recommendation, but in the end you have to decide for yourself and also bear the* responsibility *yourself. It would have been a huge relief if someone had said: OK, now do this and I’ll take responsibility.”*

*(CW 14)*

### 3.5. Main Theme 2: Isolation

#### 3.5.1. Sub-Theme: Residents’ Coping Strategies

Residents handled the pandemic and their life in a shielded environment mostly by relying onto coping strategies they had developed throughout their life experiences, especially during their deprived childhood and youth, but also through faith. However, acceptance and coping strategies varied depending on background, pathology, and cognitive ability ranging from apathy and acceptance to anger and activism. People with dementia were a particularly vulnerable target group, for whom the isolation was especially difficult. With regard to isolation and associated loneliness it has to be underlined, however, that loneliness is common among older people in institutional settings [19], while with the pandemic it has clearly increased. Beside the visitor ban, the communal and in-house activities and exchanges were interrupted as well, creating further isolation and hampering the social connectedness of residents.

*“For me, that was the worst time. I have already experienced several bad times, but being locked up, […] beats everything.”*

*(Resident 22)*

*“I had to accept it. If there is no other way. They weren’t allowed in, I wasn’t allowed out, so I had to accept it.”*

*(Resident 16)*

*“[…] that was certainly bad. But maybe not for everyone, it was very different. […] It was not a tragedy for everyone. But for some it was.”*

*(GP 20)*

*“In general, they have become clearly more apathetic.”*

*(GP 24)*

Overall, health crises appear as complex, dynamic phenomena with different phases and cumulative processes. The different phases of the pandemic were associated with different coping strategies, reactions, and acceptance behavior: while in the beginning all four groups generally reacted with resilience and understanding for restrictions, acceptance decreased as the pandemic fatigue increased. Especially when other sectors opened up again, but NHs remained stuck in isolation mode, the societal disequilibrium led to incomprehension among NH residents, relatives, and professionals.

*“It went on for such an endless time. Other sectors, bars and restaurants were open, but nobody cared about us.”*

*(Resident 22)*

#### 3.5.2. Sub-Theme: Impact on the Residents’ Health Status

The prolonged isolation had an impact on the physical, psychological, and social wellbeing of the residents. Staff noticed how the impoverished environment and lack of regular social, cognitive, and sensorimotor stimulation of isolated people posed a health-damaging burden in the long run, for both well residents and mild to moderately ill patients. Relatives and professionals observed mood changes, depressive states of mind, cognitive decline, higher risk of falling, sleep disorders, anxiety, and loss of appetite. In some cases, the increasing anxiety and depressiveness required medical treatment. On the other hand, due to the isolation and IPC measures, less infections were brought into the NHs. In addition to the lack of visitors and volunteers and the impossibility to leave the NH, residents reported they missed going to the hairdresser, buying themselves the things they like with their own money, the in-house bar, going to holy mass, and the exchange with other residents and friends. Our participants observed that the residents’ health and functional status improved with the relaxation of the measures.

*“Resignation, depressive behavior, anxiety, sleep disorders. That has been noticed.”*

*(GP 46)*

*“Making the rounds in the village and buying my things. […] That’s what I miss most.”*

*(Resident 16)*

*“Above all, not being visited […] has certainly been an enormous burden […]. It is not easy to say what damage this has caused. […] But there is certainly damage done. There is no question about that.”*

*(GP 23)*

People with dementia are particularly susceptible to the indirect consequences of isolation and confinement and may not be able to understand or adapt to the new situation [20]. Overall, the progression of dementia symptoms is modulated by the environment and the detrimental effects of a shielded and low-stimulus atmosphere on cognition and activities of daily living (ADL) led to an observable worsening of behavioral and psychological symptoms within our cohort. However, the individual coping with the lockdown was strongly associated with the stage of dementia the person was in: people in advanced stages appeared—due to their severely impaired cognitive function—less affected by the visiting ban.

*“If they have dementia and are isolated, then they become even more demented, that’s a fact.”*

*(GP 24)*

#### 3.5.3. Sub-Theme: Lack of Informal Caregivers, Volunteers, and Friends

Family members are not merely visitors, but rather partners in care [21]. With ongoing isolation, care workers had to fill numerous additional roles to compensate for their absenteeism. Furthermore, interpersonal interaction, terminal care, leisure activities, and other tasks usually carried out by volunteers posed an additional workload on NH staff. While the care workers made efforts to fill in for the absent resources and became the central and only human contact for the residents, they were unable to cover all aspects and needs, especially in NHs with a low nurse–patient ratio and high infection rates among employees. As a central coping strategy, care workers appeared to intensify their brief contact with the residents: less, but more quality time. At the same time, the residents’ caregivers outside the NHs were impacted as well, as they reported emotional distress and feeling helpless, while they also showed understanding for the necessity of IPC measures—at least during the first weeks.

*“For me, that was hard to accept. That we replace the relatives, that we are now the ones who sit next to them and accompany them on their last journey, although there is actually a daughter or a son sitting outside who would be more entitled to that.”*

*(Nurse 33)*

*“I was really in a bad state. Because I didn’t know whether I would see her again or whether I would never see her again.”*

*(Relative 42)*

### 3.6. Prospective Strategies for Crisis Management in NHs

Our study illustrates that health disasters create an atmosphere of uncertainty, where care workers as responders perceive that they have been abandoned by leadership and that disaster plans are made by leaders without input from medical and nursing staff. Thus, care workers and medical professionals of NHs need to be included in the development of disaster protocols as valuable disaster responders [8]. Our study also indicates that relatives and volunteers as essential caregivers need to be incorporated into disaster management strategies for NHs. In light of this, the present study aimed to generate implications for prospective strategies based on the analysis of our results and the participants’ input regarding optimized disaster management in NHs. Overall, the emerging themes with regard to disaster preparedness encompass (i) organization and communication, (ii) resources, (iii) patient’s wellbeing and health care, and (iv) ethical frameworks of care during disasters. Table 4 provides an overview, and the findings are further elaborated within Section 4.

*“Because the damage is done […] and we don’t want to have that a second time.”*

*(GP 10)*

*“If communication is better, if the guidelines are clear, if it is easier to get an answer or at least understanding—perhaps there was not always an answer for everything—that would contribute a lot to feeling better, to feeling more secure in the situation.”*

*(GP 20)*

## 4. Discussion

COVID-19 disproportionally affects NH populations due to their high proportion of frail adults with acute care needs. Worldwide, NHs have suffered increased mortality due to the virus and collateral damage through IPC measures. As they are still recovering, the salience of ensuring safe environments for care of the most vulnerable and frail population increased [22]. The preparation to care for vulnerable populations with chronic health conditions during disasters has been identified as a key issue in disaster preparedness before [23]; however, the present study clearly indicates that NHs were not prepared to face a health crisis and mitigation and disaster management strategies were lacking. The subsequent shift from resident-centered care towards collective-protective approaches led to an emergency vacuum: implementable strategies were missing and the subsequent development of temporary, immediate, and mostly suboptimal solutions by unprepared staff led to manifold organizational and ethical conflicts. Additionally, our findings indicate that IPC measures had long-lasting effects on the health and wellbeing of residents, relatives, and professionals. Many months after the onset of the pandemic, relationship-centered care was still not back to pre-pandemic levels and negative effects were visible. While general protocols and guidelines are needed, our study also indicates the importance of individual decision-making, so that the preventive measures can be tailored on site, depending on current circumstances, structural prerequisites of NHs, and individual needs. To be able to deal with such challenges, care workers should be educated as valuable disaster responders with regard to roles and responsibilities during disasters, situational awareness, and personal preparedness.

Health crises create an increased, urgent, and rapidly changing need for communication, while information and data on the health threat is still being gathered and assessed. Various target groups have different information needs—NHs represent an important and specific audience with special needs due to the high proportion of frail residents. Accordingly, frequent information updates, guidelines, and instructions through a global channel should be central to any crisis-response management. Additionally, residents of NHs have (mostly) a family outside with whom connection should be maintained.

Especially during disasters, health care providers are required to integrate into the larger, non-medical multidisciplinary disaster response. Most health systems do not take full advantage of the potential synergy offered by collaborative strategies between different levels and institutions of health care [24]. Indeed, our study shows that integration and continuity between the different NHs, as well as between NHs and hospitals, and between primary and specialist care, must be strengthened to integrate care for vulnerable patients during disasters. To facilitate the flow of information, digital, fast, and unbureaucratic exchange should be improved.

Overall, to reduce introduction and spread of infections, each NH should adhere to general IPC measures such as vaccinations, PPE, testing, regular cleaning and disinfection of surfaces, and staff training. The lack of adequate PPE and the prioritization of hospitals with protection supplies led to insecurity and fear and was a fundamental risk to the health of employees in NHs. NHs, with their especially vulnerable target group, must be adequately prepared and equipped with PPE.

Prevailing issues from before the crisis, such as staff shortages, have exacerbated the situation [15,16,18]. Moreover, it has been noticed that in many countries NH staff were working at more than one facility, which may be problematic with regard to infection protection and quality of care [25,26]. During the pandemic, millions of low-wage essential health workers were on the front line, and the vast majority of these workers are women. Despite being essential, many participants felt overlooked and given low priority in terms of financial, moral, and protective support. Nursing shortage is a well-known and global problem and, especially during a health crisis, leads to a situation where the demand for nursing professionals exceeds the supply and consequently impacts health care. Accordingly, underinvestment in health worker education, training, wages, working environment, and management must be a public health action priority.

Especially during emergencies with severe disruption of social connectedness, daily routines, and ongoing isolation, strategies must be developed to address mental and psychosocial needs for all people involved. These strategies can include, for instance, training and education related to social isolation and loneliness for health care workers, regular supervision on site, development of tele-health approaches and technology to support interaction with family members and community-based networks, and employment of a psycho-geriatrician [27]. In any case, our results clearly illustrate that prolonged isolation of residents is only a suitable measure during acute emergencies and within limited periods of time, as it must always be questioned and weighed up in terms of maintaining the ethical and health-promoting aspects of each individual. Efforts must be made to ensure that NHs remain open and that visits are always allowed. Importantly, people in NHs have the right to a dignified death and palliative care, even in isolation and during disasters [28]. End-of-life care by volunteers and relatives must not be allowed to slip into “illegality” during emergencies and must remain possible during times of crisis.

Against the background of unclear legal frameworks, professionals reported fear of legal consequences. In a medical context, and especially during emergencies, making decisions on behalf of someone places a high level of responsibility on the decision-maker. Overall, moral distress occurs especially within poor ethical decision-making climates, when external constraints prevent a moral judgement from being carried out [29]. Indeed, our results underline that if, due to an emergency situation, existing ethical principles in health care are suspended, there must be guidelines for new ones. However, these cannot be developed by the health care staff in charge, but must be worked out and communicated by experts working together in an interdisciplinary way, including better malpractice insurance, developing consent forms, and better rapport concerning the dilemmas faced jointly with relatives to reduce the risk of litigation, etc. Additionally, conceptual frameworks as developed for ICUs and a positive institutional ethical climate may contribute to reduce moral distress among NH staff during disasters [30].

Our results show that measures taken to protect people in NHs risk becoming paternalistic, mainly due to lack of information, strategies, and (political) pressure. In between infection control and person-centered care, the residents’ and professionals’ experienced needs may be overridden. Since implementing a complex and dynamic process of person-centered care while at the same time providing protection from physical health threats during an emergency is associated with risks, involving all stakeholders remains a central pillar of shared decision-making—especially when the threat is not merely temporary, but has become a chronic crisis. In order to ensure the will of residents is respected even during emergencies, advance directives for medical decisions by means of known strategies (e.g., living will, patient’s provision) should be given and validated during disasters. Overall, residents of NHs and their representatives should be involved in voicing their needs and their wishes in decision-making processes affecting their everyday life.

### Limitations of the Study

There are some limitations to this study that should be taken into account. Due to strict IPC measures, we were only given access to six residents, and not—as planned—ten. Additionally, we only included residents without cognitive impairments; the perspectives of people with dementia were therefore only reported through health professionals and residents. Overall, further studies are needed to fully develop disaster protocols and strategies in detail, as our data provides only implications for future strategies.

## 5. Conclusions

Our study clearly indicates that key failings with regard to disaster management in NHs include gaps within the organizational framework, lack of emergency strategies, inadequate communication, missing guidance, and lack of resources and structural support leading to multiple challenges and failures on all fronts. Additionally, the isolation measures imposed upon NHs led to ethical dilemmas and detrimental consequences for the residents. Our survey underlines the need for individual risk assessment and a balance between containment of the virus and deleterious effects of IPC measures. Without disaster preparedness protocols and education, as well as support in decision-making during disasters, professionals in NHs are hardly able to cope with emergency situations. To make NHs more resilient to unexpected public health events, it is of urgency to invest in building back more resilient NH systems.

## Figures and Tables

**Table 1:** Sample description: relatives and residents.

Category ^1^	Age	Sex ^2^	Residents’ Duration of Stay in NHs (in Years)
Relatives/informal caregivers entry 2	54	F	5
	63	F	2.75
	66	F	1.5
	50	F	2
	74	F	1.5
	48	F	4.5
	47	M	4.5
	64	F	5
	56	F	5
	65	F	0.25
	31	F	3
	64	F	4
	63	F	3.5
	68	F	4
	54	F	13
	65	F	4.5
Residents	79	F	4
	88	F	7
	86	F	0.5
	82	F	1.5
	72	M	4
	89	F	0.5

^1^ Due to rigorous data protection and joint controllership agreement, assignments to codes are not given. The order of the participants within the table is random. ^2^ F, female; M, male.

**Table 2:** Sample description: care workers and GPs.

Category ^1^	Age	Sex ^2^	Profession (Level)	Professional Years
Care workers	37	F	Nurse	6
	40	F	Nurse	10
	56	F	Care assistance	1
	53	F	Nurse (DOP)	10
	51	F	Nurse (DOP)	8
	46	F	Care assistance	15
	45	F	Nurse (DOP)	14
	21	F	Social care worker	2
	29	F	Social care worker	6
	29	F	Nurse (DOP)	2
	55	F	Social care worker	30
	50	F	Social care worker	10
	48	F	Social care worker	11
	47	F	Nurse	16
General practitioners	67	F	Medical lead	40
	53	F	Medical lead	6
	36	M	Medical lead	4
	61	M	Medical lead	25
	53	F	Medical lead	17
	67	M	Medical lead	5
	31	M	Medical lead	0.5
	43	M	Medical lead	0.25
	46	F	Medical lead	1.5
	38	M	Medical lead	1

^1^ Due to rigorous data protection and joint controllership agreement, assignments to codes are not given. The order of the participants within the table is random. ^2^ F, female; M, male.

**Table 3:** Emerging themes and sub-themes from the interviews with residents, care workers, relatives, and GPs.

Main Theme	Sub-Theme
Suspending of established care principles	Lack of strategies
	Immediate strategies
	Organization and communication
	Professional and private burden
Ethical dilemmas	Self-determination versus community welfare
	Responsibility and fear of legal consequences
Isolation	Residents’ coping strategies
	Impact on the residents’ health status
	Lack of informal caregivers, volunteers, and friends

**Table 4:** Implications for disaster management strategies in NHs.

Organization and communication	General hygienic measures	NHs should adhere to general IPC measures such as vaccinations, PPE, testing, regular cleaning and disinfection of surfaces, and staff training.
	Tailored communication	NHs need a central, accessible, timely, credible, and understandable reference system for professional and organizational support. Communication with relatives must be maintained.
	Medical lead	NHs need an attending physician with supervisory and clinical responsibilities and this position should never be vacant.
	Collaboration between NHs and hospitals	Integration and continuity between NHs and hospitals, between primary and specialist care facilitate health care choices and strengthen integrated and multi-sectoral care for vulnerable patients in NHs.
	Collaboration between NHs	Well-established strategies and individually developed concepts within specific NHs should be made available to other NHs.
	Digital and accessible communication	Digital, fast, and unbureaucratic exchange should be improved to strengthen and ease communication.
	Individual and tailored decision-making on site	Individual room for decision-making within the different NHs must be given, so that the preventive measures can be tailored on site, depending on current circumstances, structural prerequisites, and individual needs.
	Disaster management strategies	Disaster management protocols must be developed and staff educated.
Resources	Information and knowledge	Care workers should be educated in disaster response with regard to roles and responsibilities during disasters, situational awareness, and personal preparedness.
	Material resources and PPE	NHs must be prepared and equipped with adequate PPE.
	Human resources	Underinvestment in health worker education, training, wages, working environment, and management must be tackled as an international public health action priority.
Residents’ wellbeing and health care	Patient-centered care	An individual’s specific health needs including both physical comfort and emotional wellbeing should be respected during disasters. The implementation of IPC measures harming individual health needs must always be questioned and weighed up in terms of maintaining the ethical and health-promoting aspects of each individual.
	Addressing mental and psychosocial needs	Development of strategies to address mental and psychosocial are needed. These strategies can include training and education related to social isolation and loneliness for health care workers, development of tele-health approaches and technology to support interaction with family members and community-based networks, and employment of a psycho-geriatrician.
	Isolation as a temporary measure	Isolation as a preventive measure can only be a suitable measure during acute emergencies and within limited periods of time. Even during disasters, efforts must be made to ensure that NHs remain open, and visits are always allowed.
Ethical framework	Developing ethical considerations	If, due to an emergency situation, existing ethical principles in health care are suspended, guidelines for new ones must be developed by interdisciplinary experts (on local–international levels) and made accessible to the health care staff in charge.
	Involving the involved	Residents of NHs and their representatives should be involved in voicing their needs and their wishes in decision-making processes affecting their everyday life.
	Advance directives for medical decisions	In order to ensure the will of residents is respected even during emergencies, advance directives for medical decisions by means of known strategies (e.g., living will, patient’s provision) should be given and validated during disasters.
	Dignity at the deathbed	People in NHs have the right to a dignified death and palliative care, even in isolation. End-of-life care by volunteers and relatives must remain possible during disasters.

## Data Availability

Data cannot be shared publicly because of ethical reasons. However, they are available upon reasonable request from the Institute of General Practice and Public Health, Claudiana (contact via Barbara.plagg@am-mg.claudiana.bz.it).
